# Pertussis in Young Infants Throughout the World

**DOI:** 10.1093/cid/ciw550

**Published:** 2016-11-02

**Authors:** James D. Cherry

**Affiliations:** Department of Pediatrics, David Geffen School of Medicine, University of California, Los Angeles

**Keywords:** pertussis, *Bordetella pertussis*, Tdap, DTaP, leukocytosis with lymphocytosis

## Abstract

In typical pertussis in young infants, the child will appear deceptively well; he or she will have coryza, sneezing, and a mild cough. There is no fever. This progresses to gagging, gasping, eye bulging, bradycardia, cyanosis, and vomiting. There is leukocytosis with lymphocytosis and apneic episodes. Deaths relate to leukocytosis, pulmonary hypertension, and pneumonia. The source of pertussis in young infants is most often a family member with cough illness that is not recognized as pertussis. Diagnosis is based on culture/polymerase chain reaction and leukocytosis with lymphocytosis. Treatment depends on macrolide antibiotic therapy and intubation, with assisted ventilation and oxygen. Prevention is based on prophylactic macrolide treatment, immunization starting at 6 weeks of age, and immunization of all pregnant women in the second or third trimester.

Illness due to *Bordetella pertussis* is significantly different clinically from illnesses caused by most other infectious agents [1–4]. *Bordetella pertussis* can cause severe disease and death but the illness is noninflammatory in nature, except when there is a concomitant or secondary bacterial or viral infection. Pertussis in young infants is frequently severe, and deaths are common [3, 5–9].

The bacterial cause of pertussis, *B. pertussis*, was isolated in the laboratory in 1906 [10]. This led the way for development of vaccines and their subsequent routine use in the 1940s and 1950s [11–14]. Universal vaccine use led to a dramatic decline in the incidence of reported pertussis and in pertussis deaths. However, the circulation of *B. pertussis* has not been controlled by immunization, and young infants continue to be at risk for severe pertussis and pertussis deaths.

## CLINICAL CHARACTERISITCS OF *BORDETELLA PERTUSSIS* INFECTION IN YOUNG INFANTS

The clinical spectrum of *B. pertussis* illness in young infants extends from a trivial illness to severe illness resulting in death [1, 3–9, 11, 13–15]. This spectrum is influenced by many factors, including the presence and magnitude of transplacentally acquired antibodies to *B. pertussis* antigens, the sex of the infant, the age and weight of the infant at the time of exposure, the concentration of the bacterial exposure, and whether or not the infant was breastfed.

In classic illness, the infant will look deceptively well; he or she will have coryza, sneezing, and mild cough. Most importantly, there is no fever. This constellation of rather trivial symptoms often leads the physician away from realizing the potential for an upcoming serious illness and from doing further diagnostic study. What would be the paroxysmal stage in older children is characterized by lack of fever, gagging, gasping, eye bulging, bradycardia, cyanosis, and vomiting. Most young infants will have leukocytosis with lymphocytosis. During this paroxysmal stage, there will be apneic episodes at the end of a coughing fit, and these apneic episodes may result in seizures. During paroxysms there is respiratory distress, but once the fit is over there will be no distress, and physical examination of the chest will be normal. In young infants, primary *B. pertussis* pneumonia may occur and this may lead to continued respiratory distress. It is important to note that wheezing is not a manifestation of pertussis unless there is a concomitant or secondary viral infection. Children with severe, potentially fatal illness will develop pulmonary hypertension and pneumonia and will have rapid pulse and respiratory rates. Death is associated with hypotension and organ failure. It is important to note that although apneic episodes are frightening, they do not cause death. However, the hypoxia associated with an apneic episode may be a causative factor in later epilepsy and subsequent intellectual impairment [16, 17].

Similar to the clinical spectrum, the duration of illness is influenced by many factors; the most important factors are whether the mother received tetanus, diphtheria, and pertussis (Tdap) immunization during the pregnancy and whether the infant received diphtheria, tetanus, and acellular pertussis vaccine (DTaP). The majority of data relating to the duration of illness have been reported in hospitalized infants.

In a study done in Germany 20 years ago, it was noted that 71% of 101 infants <6 months of age have a cough illness duration of >4 weeks [18]. In another study in Germany (the controls in a vaccine efficacy study), it was found that the median duration of cough was 47 days [19]. In a recent Californian study of 100 hospitalized severe cases in infants ≤120 days of age, the median number of days in the hospital was 13 with a range of 1–200 days (Cherry, unpublished data).

## PAST AND PRESENT EPIDEMIOLOGY

### Cycles of Pertussis

In the cycles of reported pertussis in the prevaccine era, rate increases occurred every 2–5 years [1, 4, 12–15]. This cyclic pattern continued to occur in the whole-cell pertussis vaccine era and is still occurring in the present acellular vaccine era in countries that switched to this vaccine. The continuation of the same cyclic pattern today as that which occurred in the prevaccine era is different from that seen with other vaccine-preventable diseases [20]. When disease and the circulation of the infections agent are both curtailed, the interepidemic period lengthens. Because that has not happened with pertussis, we know that *B. pertussis* is circulating today in a manner similar to that which occurred in the prevaccine era.

In contrast with many other infectious diseases, immunity following *B. pertussis* infection or vaccination is not long lasting [21–23]. Therefore, infection and illness are occurring and reoccurring in persons of all ages. The infection rate in adolescents and adults is about 6% a year, and the cough illness rate is >507 per 100 000 population [12]. Reported pertussis is only the tip of the iceberg. Rates of reported pertussis are 40- to 160-fold less than actual illness rates. Asymptomatic infections are 4–22 times more common than symptomatic infections.

### Rates of Reported Pertussis

In the prevaccine era in the United States, the average yearly rate of reported pertussis was 157 per 100 000 population [14]. With the routine use of whole-cell pertussis vaccines, this rate was reduced to <1 case per 100 000 population in the 1970s. In the prevaccine era, 85%–90% of reported cases occurred in children between 1 and 10 years of age. Only 7%–11% of the reported cases were recognized in infants, and reported adult cases were <3%. In the early whole-cell pertussis vaccine era, 54% of reported cases were noted in infants [14]. In more recent years, an increasing number of cases has been reported in adults [21]. This varies by country, which reflects awareness of pertussis in adults rather than true difference between countries.

The low number of cases in infants in the prevaccine era is probably an artifact as infant death causes were often diagnosed as other respiratory illness (ie, influenza, pneumonia, bronchiolitis) [14]. In England and Wales in the epidemics that occurred in 1977–1979 and 1981–1982, I noted that about 360 pertussis death cases were diagnosed as due to other causes and not pertussis [14]. Using the same method, Nicoll and Gardner noted that many pertussis cases were diagnosed as sudden infant death syndrome (SIDS) [24].

### Deaths Due to Pertussis

In the prevaccine era, deaths due to pertussis were noted in children of all ages and also in adults [14, 25]. In the present era, virtually all pertussis deaths occur in infants <4 months of age [5–9]. In the prevaccine era, most deaths in infants were not diagnosed as due to pertussis and were signed out as deaths related to pneumonia, influenza, and bronchiolitis and other respiratory infections of young children [14].

### Source of Pertussis in Infants

Studies done in the whole-cell vaccine era indicated that the source of infection in an infant was usually an adult family member with a cough illness that was not recognized as pertussis [14, 15, 21, 26–28]. In the more recent acellular pertussis vaccine era, it was noted in one study that the main source of pertussis in an infant was an adolescent family member for whom acellular pertussis vaccine failed [29].

The greatest risk factor for pertussis in young infants is family size and extended family size [9, 30]. The larger the family and extended family size, the greater the likelihood that there is a person with a cough illness that has not been recognized as pertussis.

## PATHOGENESIS AND PATHOLOGY OF *BORDETELLA PERTUSSIS* INFECTIONS

During the last 110 years, many biologically active components of *B. pertussis* have been described [1, 3, 4, 11, 13]. Most of these antigens have been discovered in various mouse-model symptoms. However, in recent years it has been apparent to me and several colleagues that many of the findings in the mouse-model systems are not important in human *B. pertussis* infection and illness [1, 3, 4, 15]. Nevertheless, there are a number of antigens that play a role in human infections [3, 4]. Most of these antigens contribute to infection by adversely affecting the innate immune response or by facilitating attachment of the bacteria to the ciliated cells in the respiratory tract.

In contrast with the many factors that contribute to the infection process, clinical manifestations are caused by just 2 antigens: (1) pertussis toxin (PT), which causes severe disease and death in young infants, and (2) “cough toxin,” which presently awaits discovery [3, 4]. PT is an ADP-ribosylating toxin that can inactivate quinine nucleotide-binding proteins (G proteins) in humans [13]. This causes leukocytosis with lymphocytosis [3]. The “cough toxin” causes the paroxysmal cough and apnea [3].

The study of the pathology and pathophysiology of *B. pertussis* infection in humans has been based on postmortem studies. Because secondary and concomitant infections with other pathogens are not uncommon, it has been difficult to distinguish the findings caused by *B. pertussis* from the findings due to other agents. It was noted by Holmes [2] >70 years ago that pertussis was different from other severe infectious diseases in that there was no fever and no physical findings when the patient was not coughing. It was noted >100 years ago that the ciliated cells in the trachea, bronchi, and bronchioles were normal [31]. Our group confirmed this finding in 2008 [3, 4, 8].

It has been repeatedly noted that severe and fatal pertussis in young infants is associated with extreme leukocytosis with lymphocytosis [3–9, 13, 15]. In addition to leukocytosis with lymphocytosis, fatal cases in young infants will have pulmonary hypertension and pneumonia. In 2008, our group suggested that deaths occurred because of irreversible pulmonary hypertension due to aggregates of leukocytes (mature neutrophils and lymphocytes) in the small vessels in the lungs [8].

## DIAGNOSIS OF PERTUSSIS IN YOUNG INFANTS

The initial symptoms of pertussis in young infants (sneezing, coryza, and normal temperature) are such that the primary caregiver rarely considers an impending severe and possible fatal illness. The exposure in young infants is most often to a family member with a cough illness that has not been recognized as pertussis. The definitive diagnosis is by polymerase chain reaction (PCR) or culture of a nasopharyngeal sample (swab or aspirate). In general, PCR is more sensitive than culture.

An important test that should be done on all young infants who might have pertussis is a white blood cell (WBC) count with a differential percentage. Any count of >10 000 cells/µL with >50% lymphocytes must be considered as possible pertussis. A WBC count of this magnitude and percentage of lymphocytes should be repeated within 24 hours. A WBC count of >20 000 cells/µL with >50% lymphocytes should be considered as a very strong indication that the infant has pertussis.

Some young infants who have transplacentally acquired antibody to PT will not have the elevated leukocyte findings; they will have cough and apnea, however. Because young infants with pertussis can have rapid deterioration, they should be admitted to a hospital where an intensive care unit (ICU) is available.

## TREATMENT OF PERTUSSIS IN YOUNG INFANTS

Our group in California (consisting of epidemiologists at the California Department of Public Health and pediatric infectious diseases specialists) has been carrying out studies on infant pertussis for >6 years [5–7, 9]. We have described factors that are associated with pulmonary hypertension and death. These factors are as follows: high and rapidly rising WBC count with lymphocytosis, heart rate >170 beats per minute, respiratory rate >70 breaths per minute and onset of pneumonia within 5 days of disease onset, lower birth weight, premature birth, early age of onset of illness, decreased pulse oxygen saturation, seizures, encephalitis, receipt of steroids, receipt of nitric oxide, intubation, receipt of exchange transfusion, and extracorporeal membrane oxygenation (ECMO) therapy [5, 9]. Obviously, many of these factors relate to treatment. Sorting out which factors actually increase the risk of death is difficult. In one study, multivariate regression analysis of illness characteristics noted that low birth weight, very high WBC count, and pulmonary hypertension correlated with death [9]. Similar analysis of treatment characteristics indicated that intubation and nitric oxide treatment correlated with death. In a classification tree analysis, a WBC count of ≥46 000 cells/µL and a birth weight of <2821 g were indicators for death.

Our group's ongoing study findings (unpublished data) suggest to us that the usual treatments for nonpertussis pulmonary hypertension (nitric oxide, ECMO) and other ICU care (steroids) may be detrimental in the treatment of pertussis pulmonary hypertension. Therefore, it is my opinion that the mainstay of present ICU care should be intubation, assisted ventilation if necessary, and the administration of oxygen.

Our group would also recommend exchange blood transfusion for high and rapidly rising WBC count. This treatment has been useful in many individual cases. In addition to lowering the WBC count, it will decrease PT in the blood, which might lower the pulse and respiratory rates. Unfortunately, exchange transfusion needs to be carried out before cardiogenic shock or organ failure has occurred [7].

Of most importance in the treatment of pertussis is the administration of a macrolide antibiotic [9]. This should be done immediately in presumptive cases without waiting for a confirmatory PCR result.

## PREVENTION OF PERTUSSIS IN YOUNG INFANTS

In 2016, the mainstay of preventing pertussis in young infants is pertussis immunization and the use of prophylactic antibiotics (usually a macrolide) in an exposed infant [9].

Acellular pertussis vaccines are less reactogenic than whole-cell pertussis vaccines, but their effectiveness and duration of protection are less than that of whole-cell vaccines [32]. Therefore, it is my recommendation that all countries presently using whole-cell vaccines continue to do so. The primary schedule for whole-cell pertussis vaccination is at ages 6, 10, and 14 weeks.

In countries using acellular vaccines, it is my recommendation that the first dose of the primary series be given at 6 weeks of age and subsequent doses given at 4 and 6 months of age.

Of most importance today, in all countries, is the administration of Tdap to all pregnant women, with each pregnancy, during the second or third trimester (before 36 weeks’ gestation) [33, 34]. Maternal immunization along with administration of the first vaccine dose to the infant at 6 weeks of age, if implemented, will prevent all pertussis deaths and most severe cases of infant pertussis.
